# Interaction between physiological and subjective states predicts the effect of a judging panel on the postures of cellists in performance

**DOI:** 10.3389/fpsyg.2014.00773

**Published:** 2014-08-07

**Authors:** Satoshi Endo, Kristina Juhlberg, Adrian Bradbury, Alan M. Wing

**Affiliations:** ^1^Chair of Information-Oriented Control, Department of Electrical Engineering and Information Technology, Technische Universität MünchenMunich, Germany; ^2^SyMoN Lab, School of Psychology, University of BirminghamBirmingham, UK; ^3^Hörcentralen, Hälsa och Habilitering, Landstinget i Uppsala länUppsala, Sweden; ^4^Department of Clinical Psychology, School of Psychology, University of BirminghamBirmingham, UK; ^5^Royal Academy of Music, University of LondonLondon, UK

**Keywords:** motor control, music, anxiety, electrodermal activity, cardiac response

## Abstract

This study investigated the effect of a panel of judges on the movements and postures of cellists in performance. Twenty four expert cellists played a short piece of music, to a metronome beat, in the presence and absence of the panel. Kinematic analyses showed that in the presence of the panel the temporal execution of left arm shifting movements became less variable and closer to the metronome beat. In contrast, the panel's presence had no reliable effect on their spatial accuracy. A detailed postural analysis indicated that left elbow angle during execution of a given high note was correlated with level of heart rate, though the nature of this correlation was systematically affected by the relevant participant's subjective state: if anxious, a higher heart rate correlated with a more flexed elbow, if not anxious then with a more extended elbow. Our results suggest a change in physiological state alone does not reliably predict a change in behavior in performing cellists, which instead depends on the interaction between physiological state and subjective experience of anxiety. This highlights a need to distinguish performance anxiety from physiological arousal, to which end we advocate currency for the specific term *performance arousal* to describe heightened physiological activity in a performer.

## 1. Introduction

Social phobia has one of the highest prevalence rates in psychiatric disorders with a report suggesting that more than 10% of the population are affected during their lifetime (Kessler et al., 2005). Anxiety, in particular, has been defined as an emotion which may manifest itself as a subjective experience, as physiological arousal and as behavioral expression (e.g., Lazarus, 1991). Performance anxiety (PA) is a commonly reported experience for musicians and the term Music Performance Anxiety (MPA) is used in reference to musicians who experience a severe degree of fear during or in anticipation of public performance (Steptoe and Fidler, 1987; Wesner et al., 1990; van Kemenade et al., 1995). As with PA, the research on MPA largely identifies three symptomatic components comprising: changes in cognitive/somatic state, physiological arousal and behavior (Craske and Craig, 1984; Kenny, 2009). The cognitive/somatic state is associated with the subjective experience of negative thoughts such as worry and fear about public performance (Wells, 1997). This subjective state of musicians has typically been assessed using questionnaires quantifying experiences such as internal dialog regarding performance evaluation and psychological reactions to negative situations (e.g., Steptoe and Fidler, 1987; Kenny, 2009). The physiological component of anxiety has been evaluated in terms of electrodermal activity (Vetrugno et al., 2003), cardiovascular response (Friedman, 2007), and respiration (Martinez et al., 1996). Changes in these autonomic functions are linked with heightened cortisol and epinephrine levels in the bloodstream mediated by the hypothalamus-pituitary-adrenal system in response to a stressful stimulus (Tsigos and Chrousos, 2002). It is speculated that this change in physiological state prepares the body for immediate response to potentially threatening stimuli, often described as the fight or flight response (Jansen et al., 1995). A handful of studies have measured physiological responses when participants have performed in front of an audience. Fredrikson and Gunnarsson (1992), for example, found significant increases in heart rate and neuroendocrine activation compared with when they performed without an audience. These participants also rated themselves more distressed before audience, though no relationship was found between self-rated distress and physiological responses.

Out of the three components of MPA, behavior is the least well understood in the literature. Behavioral theories of anxiety disorder state that anxiety is associated with appraisals of danger which typically result in behaviors such as avoidance and so called safety-behaviors (Wells, 1997). For example, Cox and Kenardy (1993) reported that anxiety in music performance in some instances leads to avoidance of performance situations. Furthermore, Bögels and Mansell (2004) argued that anxiety is associated with hypervigilance to a potential threat which leads to behavioral modifications. From this perspective, musicians performing before an audience may experience postural changes such as upper limb withdrawal (De Silva and Bianchi-Berthouze, 2004) and a generalized state of co-contraction or “freezing” (Azevedo et al., 2005), symptoms associated with anxiety/fear. To date, the behavioral element of MPA has been assessed in terms of motor task success (i.e., performance quality) and/or anxiety behavior checklists. For instance, Craske and Craig (1984) employed two expert judges to rate various characteristics of pianist performance such as their touch, phrasing, pitch, and rhythm. In parallel, they used videotape to assess a timed checklist of overt indications of MPA such as hand and leg trembles and stiff postures. A few studies have rated and compared performance in musicians designated as high-anxious and low-anxious participant groups and have found the performance quality to be lower in the more anxious group as rated by judges (e.g., Craske and Craig, 1984; Fredrikson and Gunnarsson, 1992). Interestingly, however, Fredrikson and Gunnarsson (1992) reported that the quality rating purely based on the acoustic performance was conversely better for the high-anxious group, and the authors suggested that the visual cues about task-irrelevant behavioral characteristics such as body posture may have contributed to the way in which the public performance of the high-anxious group was perceived by the judges to be worse.

While previous research has depicted how each component of MPA is affected when performers experience anxiety, their relationships remain unclear. Historically, Lang (1971) argued that the three components of fear reactions (i.e., verbal, physiological and behavioral) are interactive but also partially independent. These components are capable of responding differently at any one point in time, with covariation defined as concordant, and their independent variation as discordant (Rachman and Hodgson, 1974). In their review of anxiety assessment methods, Lawyer and Smitherman (2004) reported that Lang's tripartite model was most widely employed from the mid- to late 1970's up until 1990. However, for at least the next decade, such multi-system assessment was in decline, with researchers instead relying on either one or two of the three components for their analyses. Nevertheless, two theoretical frameworks in particular offer multidimensional models of anxiety which account for the complexity in drawing relationships between the three components of anxiety: the conscious processing hypothesis (Masters, 1992) and the processing efficiency theory (Eysenck et al., 2007). In general, these theories suggest that performance is impaired when the state of anxiety limits the level of information processing in the central nervous system either due to attentional shift to concerns associated with the anxiety or to reduced attentional resources for task relevant information (Masters, 1992; Eysenck et al., 2007; Coombes et al., 2009). For instance, Masters' conscious processing hypothesis (Masters, 1992) proposes that in an anxious state the performer engages in overt monitoring of his/her movements and that this process may disrupt the automatic execution of well-learned movements. On the other hand, the processing efficiency theory predicts that the cognitive load is increased in the state of anxiety and this compromises the performance efficiency (Eysenck et al., 2007). Such theories predict that reduced cognitive capacity leads to a decline in performance quality (Mullen et al., 2005; Hardy and Hutchinson, 2007) and a regression of performance standard to earlier stages of skill acquisition (Pijpers et al., 2003; Wan, 2005). A defining characteristic of the conscious monitoring hypothesis is that the anxiety primarily influences the motor efficiency, and so the performer can at least compensate for the effects of anxiety by raising levels of effort.

Overall, studies investigating sports performance have indicated that behavior is an important variable in the anxiety-performance relationship, and specifically that heightened subjective and physiological arousal are associated with behavioral changes that may, in turn, impair motor performance (e.g., Pijpers et al., 2003). From a motor control perspective, the richness of activity involved in cello playing affords a unique theater of research wherein details of movements affected by MPA may be investigated. A performance by a string player requires precise upper body motor coordination in time, space and force to create a desired sound, which is acquired through intense practice (Ericsson et al., 1993). Motor control research on string players is a well-established topic in the literature, showing for example that the accurate and efficient performance of music is achieved by exploiting the degrees of freedom in the arm in a way that it varies with the player's level of experience (Winold and Thelen, 1994; Verrel et al., 2013b). In particular, a study by Verrel et al. (2013b) shows that players tend to reduce the use of the distal joint (i.e., wrist) in bowing technique with advances in the standard of performance (c.f. Konczak et al., 2009). Furthermore, Chen et al. (2006) reported the remarkable ability of cellists to maintain pitch accuracy in time and space when executing large left hand shift motions along the fingerboard.

Thus, we investigated how the spatial and temporal coordination of pitch control in cellists is affected by the stress of performance in public, to better understand the relationship between the three symptomatic components of MPA. The behavioral variables included in the study were chosen on the basis of anecdotal spoken evidence from cellists as well as literature investigating anxious and/or fearful behavior. The present study collected self-reported levels of anxiety, measured heart rate and observed behavioral responses by monitoring the spatio-temporal control of upper body movements in participants when performing alone and in front of an audience. Although our PA literature review included many behavioral studies, we did not discover instances of behavior being assessed as a continuous and directional variable, measurement that would enable correlational patterns between the components of MPA to be investigated more thoroughly. Therefore, we chose to use motion capture technology to record translational and angular movement data in an attempt to move PA research in this new direction. In summary, we set out to investigate interaction between the three symptomatic components of MPA, using the novel methodological approach of motion capture in the case of assessing behavior. In reference to previous research, we expected to find some discordance between the three elements of MPA, but aimed to reveal, owing to the accuracy and detail afforded by our behavioral measure, patterns of interaction hitherto unrecorded.

## 2. Materials and methods

### 2.1. Participants

Twenty four advanced to concert-level cellists volunteered in this study. At the time of testing, 16 of them were student musicians (26.6 ± 5.5 years old) and 8 were professional musicians (33.5 ± 5.4 years old). The mean cello experience was 14.6 ± 2.8 years for the student musicians and 21.75 ± 2.75 years for the professionals. There were 15 female and 9 male participants. All participants reported to be in good health at the time of testing. Two participants reported taking prescription medication, one for asthma and the other for diabetes. Due to the possible link of diabetes with the cardiovascular autonomic neuropathy which leads to abnormal heart rate variability (Kudat et al., 2006), the latter participant was excluded from the heart rate analyses. The study was approved by the School of Psychology Research Ethics Committee at Birmingham University and informed consent was obtained from all the participants prior to testing. Participants were paid for their time upon completion of the study.

### 2.2. Stimuli and apparatus

#### 2.2.1. Music piece and panel

**Figure 1:**
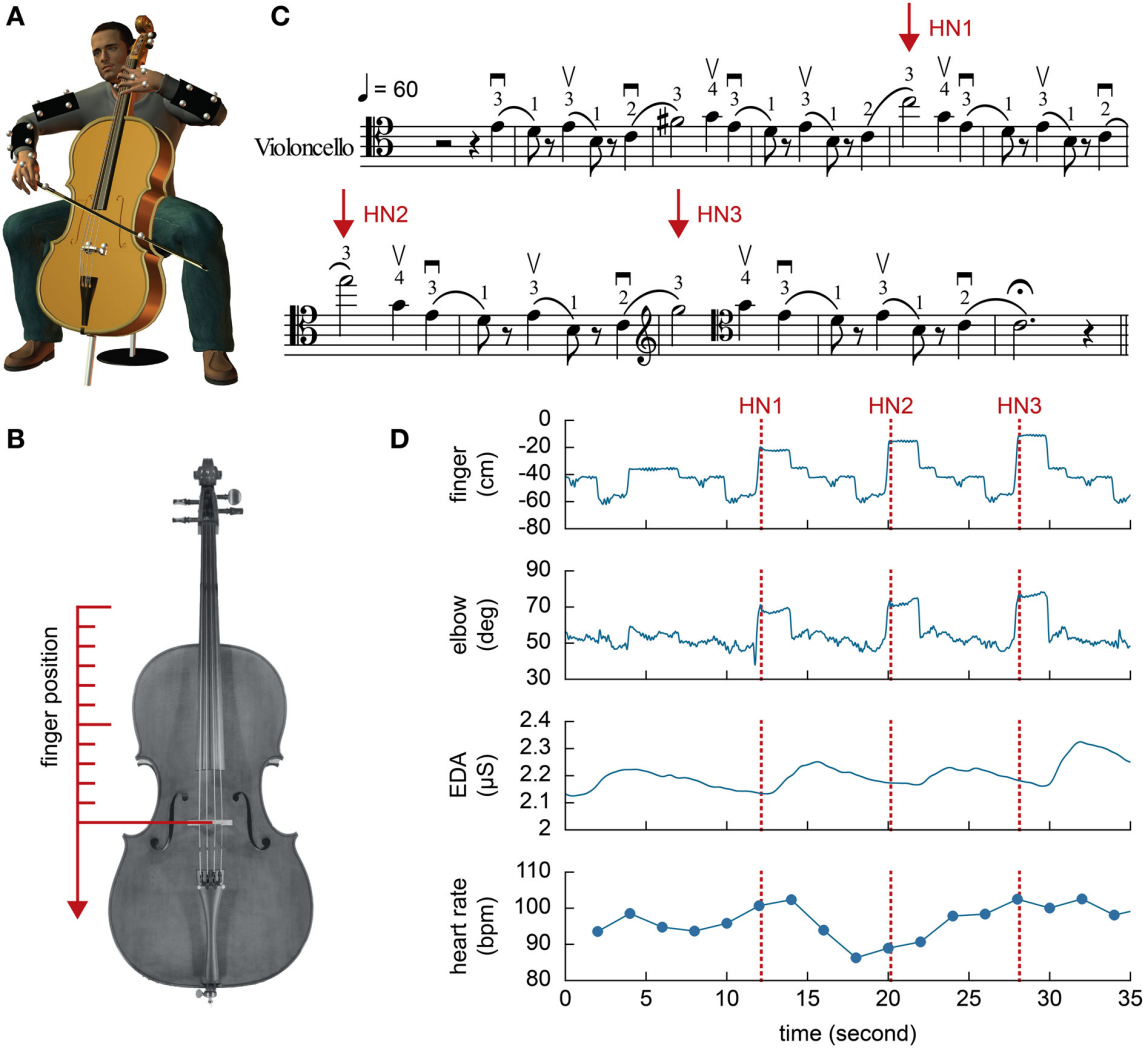
**(A)** Illustration of the experimental setup. A total of 29 small markers were attached to the upper body of the participants and the instrument. **(B)** The left hand ring finger position was transformed to align with the longitudinal axis of the instrument and the bridge on the cello was used as the center of the Cartesian coordinate. **(C)** Music score designed for the purpose of the present study. The three high notes are indicated by the arrows. Fingerings (instructions on which finger to use, where index = 1, middle = 2, ring = 3, little = 4) were specified in the score for the participants. **(D)** An example of behavioral and physiological measures from a single trial. The heart rate is converted to bpm. Zero in the finger position indicates the position of the bridge.

Participants played a piece of music based on a fragment of Brahms Double Concerto Op. 102, specifically arranged and fingered for this study by one of the authors (Adrian Bradbury), and given to each participant at least 24 h before their session. The music score was an 11-bar piece played at a tempo of 60 bpm. The piece contained three high notes (indicate by arrows in Figure 1C) placed two bars apart from each other and executed with the ring finger of the left hand. The pitch of the high notes, and therefore the distance covered by the left hand up the fingerboard to reach them, increased progressively over three levels. In an attempt to provoke stress in the participants, in the middle of the experiment, the panel of judges was introduced into the room and sat behind the table, facing the participants. The panel consisted of two of the authors. All participants knew one member of the panel (Adrian Bradbury) as he was either their colleague or on their teaching staff, but not the other (Alan M. Wing or Satoshi Endo). The experiment was conducted by another researcher (Kristina Juhlberg) and the panel had no interaction with the participants throughout the study and merely acted as observers. The participants were not informed that the judges were researchers. To further attempt to increase the performance pressure, the panel also informed the participant that his or her performance would be rated and recorded. In addition, the panel turned on the red light bulb placed in front of them to signal the start of the (sham) recording. We judged that some pressure would at least be elicited by association with genuinely stressful performance situations, hence the panel behind a desk (quasi audition situation) and the red light (quasi recording studio situation). The panel was encouraged not to make unnecessary facial expressions, or make verbal/non-verbal communications with the participants.

#### 2.2.2. Behavioral measures

A 6-camera Oqus motion-tracking system (Qualisys, Sweden) was used to record kinematics of the participants at 200 Hz. A total of 29 reflective markers were attached to the left and right upper arms and forearms (4 markers for each segment), left and right hands (×3), left ring finger, cello (×4) and bow (×2) (Figure 1A). The markers on the cello were used to define the Cartesian coordinates of the ring finger position along the length of the instrument fingerboard with the origin of coordinate aligned at the bridge on the cello (Figure 1B). In order to equalize the tempos of the participants' performances, a computer-generated metronome (60 bpm) sounded while they played the music. The analog signals of the metronome pulses were synchronously recorded with the motion data in the same computer at 1000 Hz.

#### 2.2.3. Physiological measures

The physiological state of the participant was monitored by measuring electrodermal activity (EDA) and heart rate using BIOPAC MP150 System (Biopac Systems, USA), recorded at 1000 Hz. The EDA electrodes (TSD203) were attached to the proximal phalanges of the right index and middle fingers, such that there was no interference with bowing technique. The heart rate was measured in terms of pulse pressure at the right earlobe using an infrared transducer (TSD200C).

#### 2.2.4. Subjective measures

A one-item Likert scale based on subjective units of distress was used to measure the stress experienced by the participants where 1 represented “completely relaxed” and 7 “unbearably anxious” with a 0.5 interval scale. This measure was collected before and after each trial, so providing a reflection of each participant's response to the challenges of the experiment over a finer time scale than measures offered by existing standardized questionnaires. The subjective ratings were later validated by correlating them with factors identified in two standardized questionnaires: one developed by Steptoe and Fidler (1987) and the other called “reactions to tests” (Sarason, 1984). The former questionnaire consists of 20 self-statements and coping strategies that Steptoe (1983) previously found musicians used before a performance. These statements make up six factors including *catastrophizing*, *positive thinking*, *mixed strategy*, *blasé attitude*, *realistic appraisal* and *audience sensitivity*. The latter questionnaire measures aspects of anxiety in relation to test taking. The statements within the questionnaire assess four different dimensions of test anxiety including *worry*, *tension*, *test-irrelevant thinking* and *bodily symptoms*. For the purpose of this study, the wording of the items within the questionnaire was adapted, i.e., the word test(s) was changed to performance(s), to relate to a music performance situation instead of an academic test situation.

### 2.3. Design

This study was a 3 × 3 within-subject design. The first independent variable was the presence of the Audience such that participants played the piece before the panel of judges came in (Pre-Panel), in front of the panel (Panel), and after the panel had left the room (Post-Panel). The second independent variable was the pitch of three High Notes (HNs) embedded in the music, and their relative pitch was either low (HN1), medium (HN2), or high (HN3). There were three categories of dependent variables: behavioral, physiological and subjective measures. As a preliminary analysis indicated no significant difference between the students and professionals in any of the measures used in the current study (c.f. Steptoe and Fidler, 1987), their musical levels were not considered as subgroups for the main analyses.

#### 2.3.1. Behavioral measures

Prior to analyses, the kinematic data were smoothed using a second-order bidirectional low-pass Butterworth filter with a cut-off frequency of 12 Hz. The left hand ring finger position was transformed to a coordinate system defined by the instrument, such that the closer the finger was to the pegbox of the cello in its longitudinal axis (and the lower was the note), the lower (more negative) was the spatial value (Figure 1D). This transformation was performed to express an overshoot of the fingering on a high note as a positive shift and an undershoot as a negative shift. The longitudinal positions and the times at which the ring finger reached each High Note were analyzed given that both measures directly related to the acoustic performance. Prior to testing, the finger positions for all of the three High Notes were recorded by each participant without any time constraint, and out of the musical context for calibration purposes (see 2.4). In the trials, the High Note was detected at the first instance where the velocity of the finger reached zero along the axis aligned to the strings following the high velocity movement for the shift movements up to the High Notes. The deviations of the High Note finger positions during the trials from their pre-recorded reference points were calculated as a spatial measure of the performance. For temporal measures, the time differences between the High Notes and their respective metronome beats were calculated so the positive values meant the High Notes were being placed after the metronome beats. It is important to note that both spatial and temporal measures do not necessarily relate to the quality of performance and their magnitudes could be resulted from musical interpretations. In addition the left elbow angle during the High Notes was calculated to examine the kinematics of shift movement completion.

#### 2.3.2. Physiological measures

Physiological measures consisted of the heart rate and EDA. For calculating the heart rate, the blood pressure signal was high-pass filtered using the bidirectional second order Butterworth filter (cut-off frequency of 0.5 Hz). The inter-beat intervals of each pulse were then calculated by sampling the peak time of each pulse. Analyses of the physiological signals were related not only to the Panel and High Note pitch factors but also to the time course before and after High Notes. In order to quantify within-trial variations in the physiological responses around the time of High Notes, therefore, the physiological data from each trial were subdivided into epochs of 2 seconds each, consisting of epochs “−2” (4–2 seconds before HN), “−1” (2–0 seconds before HN), “0” (0–2 seconds during HN) and “+1” (2–4 seconds after HN).

### 2.4. Procedure

At the beginning of the experiment, the reflective markers and physiological sensors were attached to the participant. The participant was then asked to sit down and to play his/her instrument to ensure that he/she was comfortable and not restricted by any of the equipment during performance. At the beginning of the experiment, two types of calibration of motion tracking data were performed. The first one concerned the joint locations of the participants. Markers were placed on the medial and lateral aspects of the wrist, elbow and shoulder joints in order that these joint positions could be reconstructed from the main experimental data sets where these joint markers were no longer present. This procedure was employed instead of directly placing the markers on the joints of the participants to minimize an obstruction of the markers on participants' movement during the main experiment. The second calibration concerned the left hand ring finger position during the High Notes, for which the participants played the three High Notes in turn and held the finger on each one for 5 seconds. These marker positions were recorded three times and the average positions were later used as reference points for data analyses in order to calculate the spatial measure of High Notes. When the participant was ready he/she was asked to indicate how anxious they felt by pointing their bow to a number on a scale between 1 and 7 printed on a board by their feet that could not be seen by the panel when they were in the room. They then played the music in time with a metronome. At the end of each trial, they were once again asked to indicate how they felt using the same scale. After 5 trials, the participants were told that the investigator would now call the panel who would judge their performance. The investigator then left the room and came back with the panel members who had been waiting in a different room. When entering the room the investigator introduced the participant and the panel members by name, and the panel was seated behind a large table in front of the participant. The panel also informed the participant that they would be recorded and turned on a red light to indicate this. A (sham) audio recording of their performance was made with the red light on to simulate a recording studio. With the panel present the participant played the music 5 more times. The panel left the room, turning the sound recording and the red light off. The participants then played 5 more trials. At the end of the experiment, the participants filled in two questionnaires, one developed by Steptoe and Fidler (1987) and the other, “reactions to tests” questionnaire, by Sarason (1984).

## 3. Results

The results section first describes the changes in the subjective experience of the participants associated with the presence of the Audience and the playing of the High Notes, followed by a description of the corresponding changes in their physiological measures. We then report kinematic measures of cello performance in terms of spatial, temporal and postural control during and after shift movements to describe an overview of the kinematic changes under pressure. Finally, the relationship between the subjective, physiological and kinematic measures is studied to demonstrate how these indices of cello performance interact with each other. For statistical analyses, an alpha level of 0.05 or less was considered as statistically significant and the Bonferroni corrections were used for multiple comparisons.

### 3.1. Subjective measure: one-item likert scale

**Figure 2:**
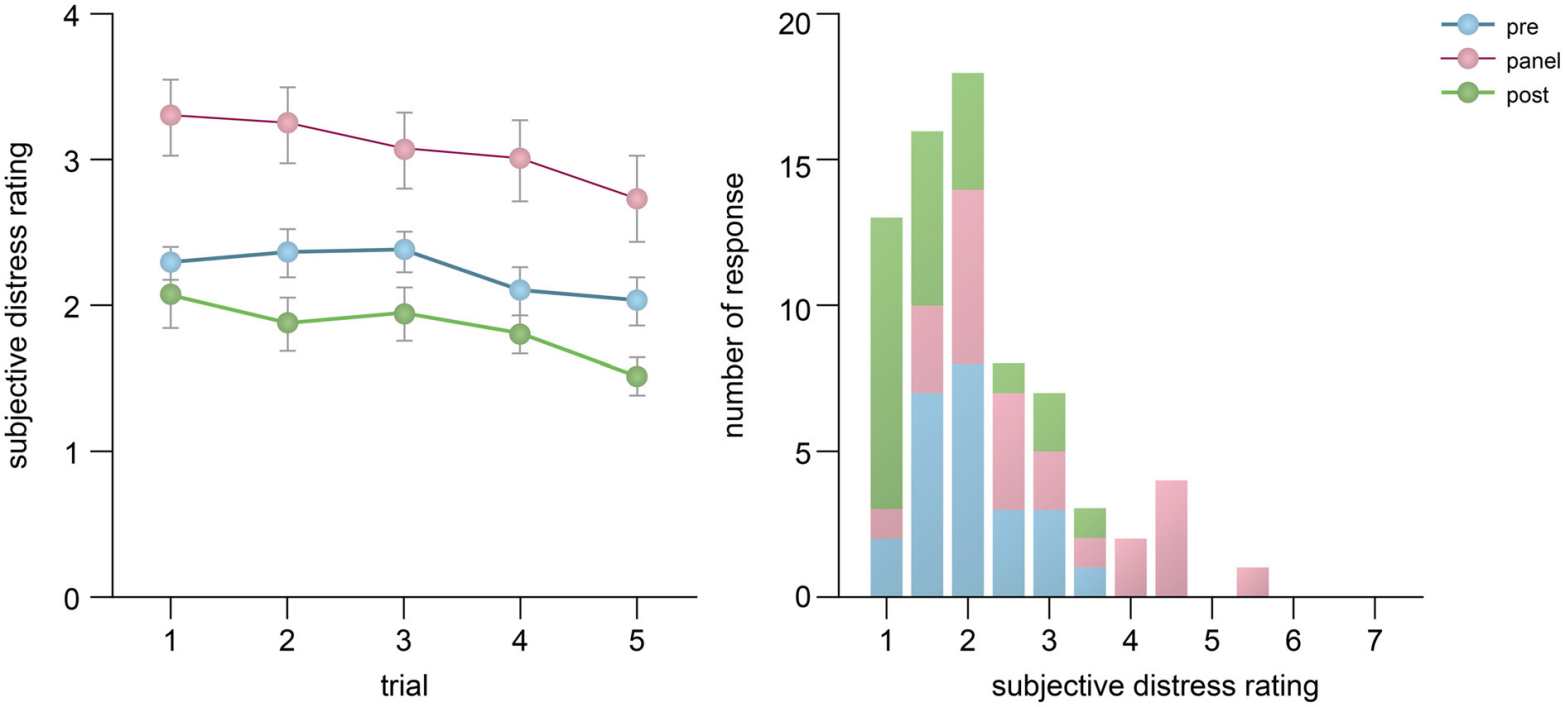
**Changes in one-item Likert scores across trials and their distribution**. The Likert scores obtained before and after a trial were averaged. The error bars represent one standard error.

In this section, we report the changes in the subjective experience of the participants associated with the presence of the Audience, repetitions of Trial (5 trials) and Time of report (before vs after a trial). The analysis indicated that the participants reported themselves to be most anxious when the Panel was present, followed by the Pre-Panel stage, and least anxious in the Post-Panel stage (Figure 2). Furthermore, the subjective rating was highest at the first trial of each condition and gradually reduced over the repetition of trials. A 3-way repeated-measures ANOVA confirmed the main effect of Audience, *F*_(2, 46)_ = 30.27, *p* < 0.0005 and a main effect of Trial, *F*_(4, 91)_ = 10.523, *p* < 0.0005. Pairwise comparisons indicated that the participants reported to be significantly less anxious in the Pre-Panel (*p* < 0.0005) and Post-Panel (*p* < 0.0005) stages than in the Panel stage. No main effect of Time of report or any interaction effects were observed (*ps* > 0.20).

Table 1 shows the correlation of subjective rating with factors in the standardized questionnaires in order to evaluate validity of the subjective rating. For the factors in the adapted “reactions to tests” questionnaire (Sarason, 1984), the analysis indicated our rating was positively correlated with *worry* and *tension*. The *catastrophizing* factor in the Steptoe and Fidler (1987) questionnaire was also positively correlated, altogether partially supporting the validity of the subjective rating used in the present study along with the established measures.

**Table 1:** **Pearson's correlations of the subjective distress rating in the present study with factors identified in standardized questionnaires (Sarason, 1984; Steptoe and Fidler, 1987)**.

**References**	**Factor**	**Correlation**	**Sig**
Sarason, 1984	Tension^*^	0.512	0.011
	Worry^*^	0.473	0.019
	Irrelevant thinking	0.278	0.189
	Bodily symptoms	0.212	0.320
Steptoe and Fidler, 1987	Catastrophizing^*^	0.464	0.022
	Positive thinking	0.082	0.704
	Mixed strategy	0.303	0.149
	Blasé attitude	0.217	0.308
	Realistic appraisal	−0.188	0.379
	Audience sensitivity	−0.003	0.988

* *Significant correlations*.

### 3.2. Physiological measures

The physiological responses were measured in terms of the EDA and heart rate, and the effect of the Audience and the High Note movements on them were studied. In order to quantify within-trial variations in the physiological responses with respect to the High Notes, the physiological responses were subdivided into four epochs of 2 seconds each, with two epochs either side of the start of each High Note. The averages of each epoch were then analyzed with the other independent variables using a three-way repeated measures ANOVA.

#### 3.2.1. Electrodermal activity

**Figure 3:**
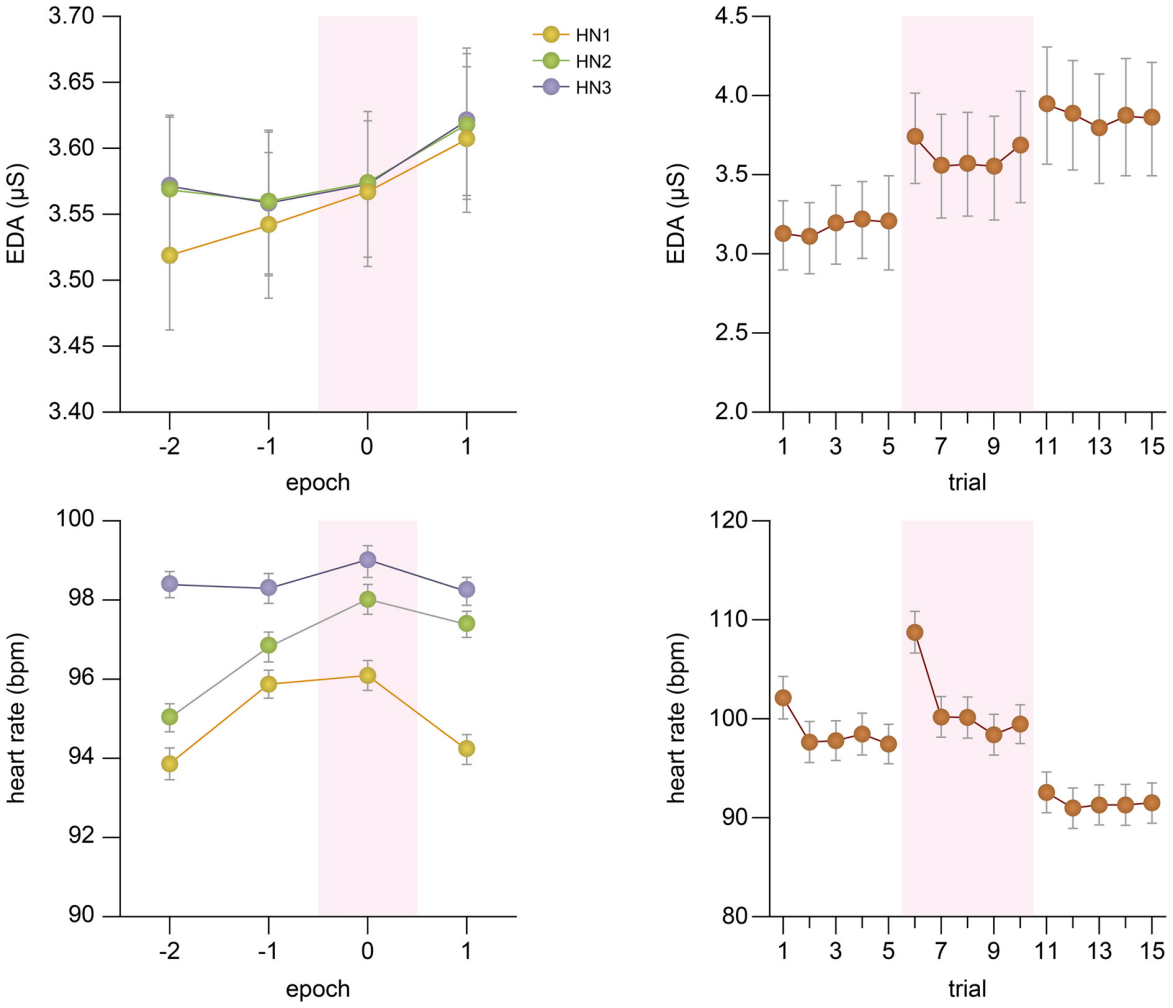
**EDA and heart rate changes within and across trials**. In the left panels, the physiological responses around the time of the high notes are depicted over four epochs of 2 seconds each. Epoch 0 is aligned with the respective metronome beat for High Notes and this is highlighted. In the right panel, the changes in the average EDA and heart rates over the course of 15 trials are shown. The Panel stage (trial 6–10) is highlighted. The error bars represent one standard errors.

Figure 3 illustrates changes in the EDA within and across trials. Overall, the lowest EDA was measured in the Pre-Panel stage. A moderate increase of the EDA was observed in the Panel stage and the highest EDA was observed in the Post-Panel stage, likely due to the accumulation of non-evaporated sweat. While the EDA was high throughout Epochs for HN2 and HN3, the EDA was considerably lower before the onset of HN1. However, a steady increase of EDA was observed for HN1, peaking at a similar level to the other HNs after the execution of the shift movement (Epoch 1). The 3-way ANOVA supported a main effect of Audience, *F*_(2, 46)_ = 3.26, *p* < 0.05. The *post-hoc* analysis indicated that the EDA was significantly higher in the Post-Panel stage than the Pre-Panel stage (*p* < 0.001). Furthermore, a main effect of Epoch was found, *F*_(3, 69)_ = 23.47, *p* < 0.0005. There was no main effect of High Note (*p* = 0.41), but an interaction between High Note and Epoch was significant, *F*_(6, 138)_ = 5.01, *p* < 0.0005.

#### 3.2.2. Heart rate

The heart rate was highest during the Panel stage and lowest in the Post-Panel stage. The heart rate also progressively increased from HN1 to HN3. In HN1, a clear increase of the heart rate was observed in synchrony with the execution of the shift movement (Epoch 0), and it immediately returned to the baseline level by Epoch 1. This modulation was significantly smaller in HN3, likely due to the ceiling effect in which the heart rate was generally much higher than the other HNs. A 3-way ANOVA indicated a main effect of the Audience, *F*_(2, 44)_ = 42.56, *p* < 0.0005. The *post-hoc* analysis showed that the heart rate during the Panel stage was significantly higher than during the other stages (*ps* < 0.05). There was also a main effect of High Note, *F*_(2, 44)_ = 39.78, *p* < 0.0005. There was an interaction effect between Audience and High Note, *F*_(4, 88)_ = 2.92, *p* < 0.03, and between High Note and Epoch, *F*_(6, 132)_ = 2.72, *p* < 0.02.

### 3.3. Kinematic measures

The kinematic measures consist of temporal, spatial and postural aspects of the shift movements in precision and cross-trial variability. Firstly, their mean changes with respect to the presence of the Audience and the High Note were analyzed using 3 × 3 repeated-measures ANOVA. Secondly, the variability (standard deviation) of the kinematic measures were log-transformed to correct for non-normal distribution and submitted to a separate ANOVA.

#### 3.3.1. Temporal control: high note arrival timing

The participants on average completed the shift movement to arrive at a High Note 139.6 ± 76.8 ms after the corresponding metronome beat in the Panel stage. The timing of the shift movement was slightly delayed in the Pre-Panel stage (157.7 ± 73.3 ms) and the largest delay was observed in the Post-Panel stage (190.6 ± 86.9 ms). Furthermore, the shift time and its variability progressively increased with the High Notes. The ANOVA indicated there was a main effect of Audience on mean temporal deviation, *F*_(2, 46)_ = 7.39, *p* < 0.003. A main effect of the High Note was also found, *F*_(2, 46)_ = 10.41, *p* < 0.0005. The pairwise comparisons indicated that the delay at HN1 was significantly less than the remaining High Notes (*ps* < 0.03). No interaction effect was observed (*p* = 0.57). A separate ANOVA also confirmed that the variability of the temporal deviation was affected by the High Note indicating that the size of the variability increased with the order of the High Notes, *F*_(2, 46)_ = 5.590, *p* < 0.001. No significant main effect was observed for the Audience (*p* = 0.58).

#### 3.3.2. Spatial control: high note fingering

**Figure 4:**
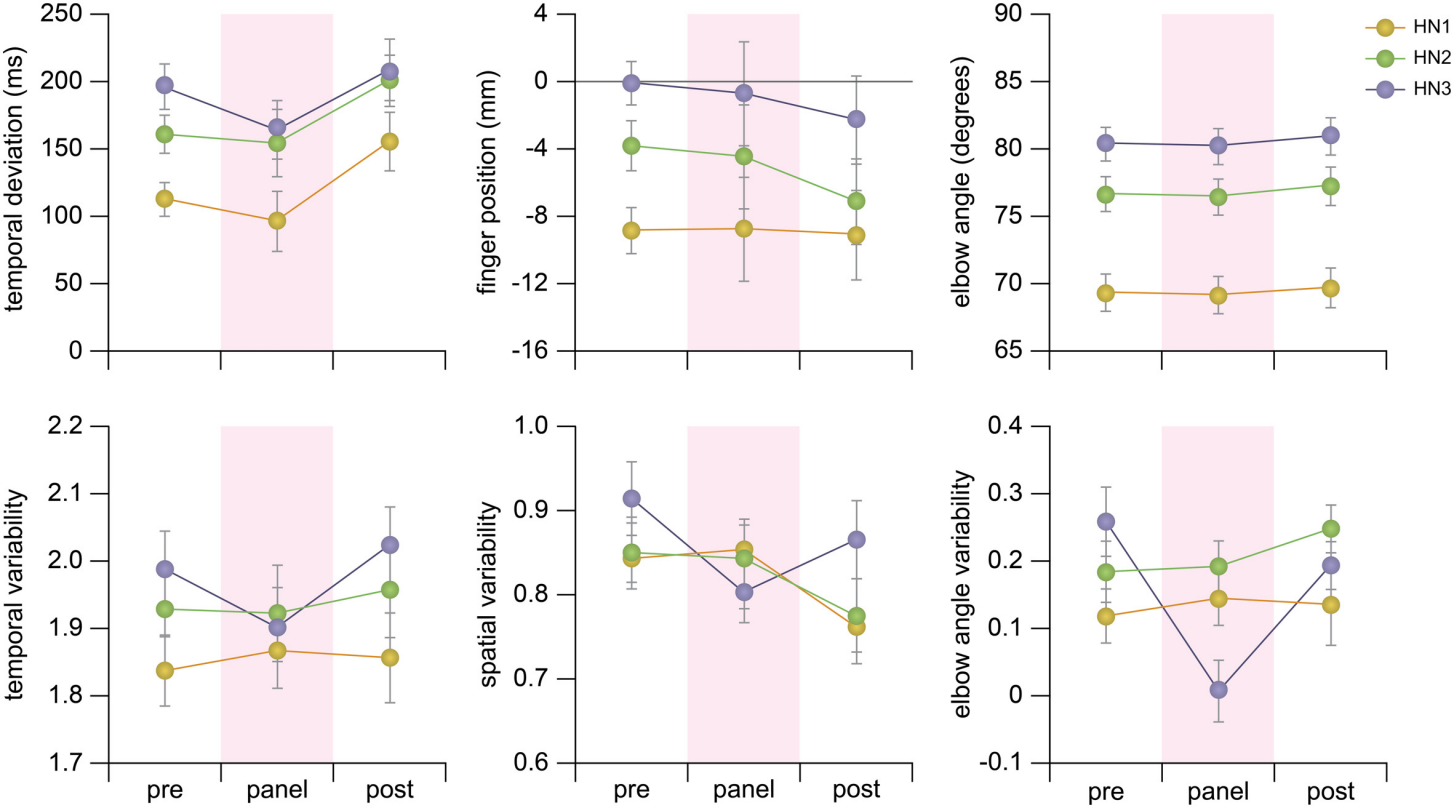
**Means and variability of behavioral measures consisting of temporal deviations of the high note, spatial(finger) deviation of the High Note, and the elbow angle at the High Note**. The standard deviation of the behavioral measures from each participant was log-transformed to correct for non-normal distribution. The error bars represent one standard errors.

In general, the participants' shift movements undershot the High Notes as represented by the negative values in Figure 4. Changes in the degree of undershoot specific to presence of the Panel were not observed but it increased from the Pre-Panel to the Post-Panel stages in the order of experimental manipulation. With respect to the High Notes, the largest undershoot was observed at HN1 and the finger position was closest to the target at HN3. The ANOVA showed that there was a main effect of Audience, *F*_(2, 46)_ = 6.04, *p* < 0.005. Pairwise comparisons revealed that there was a significant difference between the Pre- and Post-Panel stages (*p* < 0.005). A main effect of High Note was also found, *F*_(2, 46)_ = 5.14, *p* < 0.10. Pairwise comparisons confirmed that the degree of undershoot at HN1 was significantly larger than at the remaining HNs (*ps* < 0.02). No interaction effect was found (*p* = 0.22). The size of variability was not affected by the Audience (*p* = 0.12) or the High Note (*p* = 0.30).

#### 3.3.3. Postural control: left elbow angle

The left elbow angle during High Notes was smallest in the Panel stage. The angle was larger in the Pre-Panel stage and the largest in the Post-Panel stage although the effect of the Audience was not statistically reliable (*p* = 0.094). As predicted, the angle sizes were different across High Notes, *F*_(2, 46)_ = 303.91, *p* < 0.0005; the elbow angle gradually increased from HN1 to HN3. The *post-hoc* tests confirmed the differences in elbow angles in all pairings of the three High Notes (*ps* < 0.001). No interaction effect was observed (*p* = 0.889). Furthermore, a separate ANOVA indicated a main effect on Audience in the variability of the elbow angle size, *F*_(2, 46)_ = 3.565, *p* < 0.04, such that the variability in the Panel stage was significantly smaller than in the rest of the stages (*ps* < 0.05). There was no main effect of High Note (*p* = 0.08). However, an interaction between Audience and High Note was found, *F*_(4, 92)_ = 3.663, *p* < 0.01. This interaction effect was caused by the prominent variability reduction at HN3 in the Panel stage (see Figure 4).

### 3.4. Interaction between subjective, behavioral, and physiological measures

**Figure 5:**
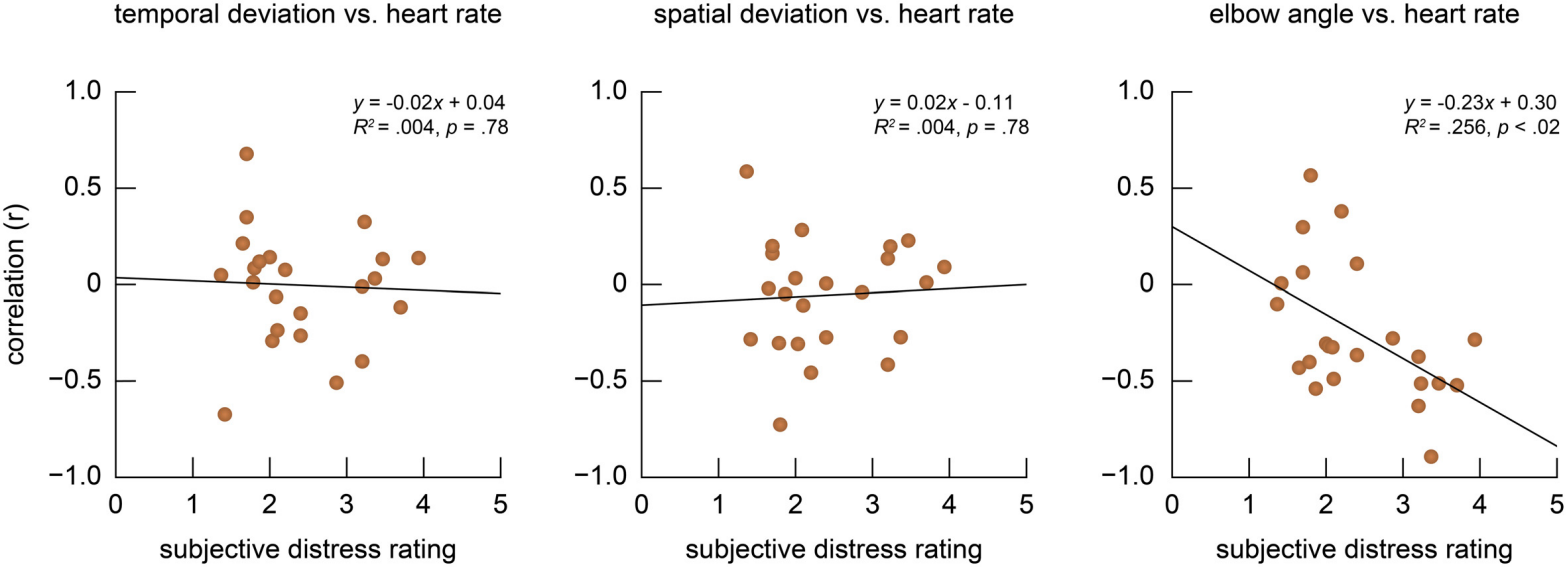
**Individual differences in the correlation between the physiological measure (i.e., heart rate) and behavioral measures in the Panel stage**. Those who reported the task as more anxiety-provoking showed negative correlation in the heart rate and elbow angle but the correlation was reversed in less anxious participants. The lines indicate a regression.

Despite the fact that a significant increase in the physiological response was observed at the Panel stage, the subjective experience of the performance varied across individuals. Thus, we studied how the individual differences in the subjective distress rating interacts with physiological and behavioral measures. Figure 5 illustrates variations of the correlation coefficients between the physiological measure (i.e., heart rate) and temporal, spatial and postural behavioral measures in the Panel stage plotted against the averaged subjective rating across participants. Although no systematic relationship was found in the temporal and spatial behaviors of the performers (*ps* = 0.78), there was a linear relationship in how the elbow angle correlated with the physiological response (*r*^2^ = 0.256, *p* < 0.02) over their distress rating. The analysis revealed that three participants showed a tendency for positive correlation (defined as *r* > 0.25) between the physiological and behavioral measures, meaning that the increased heart rate was associated with more extension of the left arm at a given high note. In contrast 17 participants showed a conversed negative tendency (*r* < −0.25). The relationship between the heart rate and the left arm posture varying with their subjective distress rating suggests that the “positive correlation group” were predominantly observed among those who reported to be less anxious during performance (mean distress score = 1.9 ±0.27) and the negative correlation group commonly reported a higher level of anxiety (mean distress score = 2.63 ±0.77). The results indicate that the relationship between the physiological and behavioral components of music performance is not stably defined but it is moderated by their subjective distress level. This observation was evident in the postural control of the performers but not in the temporal and spatial control of the shift movements.

## 4. Discussion

The main purpose of the present study was to investigate kinematic changes in cello playing when performing under pressure and their relationships with physiological and subjective responses. Specifically, we asked a group of cellists to perform individually with and without a panel of judges present. While the physiological and subjective measures indicated clear changes associated with the presence of the panel during performance, a group of behavioral measures yielded complex patterns of results varying with the task difficulty and individual differences in stress level.

For example, kinematic analyses revealed that the spatial accuracy of left hand shifting movements was not overall affected by the presence of the panel. On the other hand, the spatial accuracy and its variability were strongly affected by the location of the High Notes. Namely, the highest note (HN3) was played with the highest spatial accuracy and with least variability despite the general consensus that spatial variability and movement amplitudes are positively correlated (Schmidt et al., 1979; Meyer et al., 1988). In contrast, the delay in the time of completion of the shift movement execution progressively increased with the high notes. This negative relationship is in consistent with Fitt's law (Fitts, 1954) which describes that the central nervous system compensates between the spatial and temporal aspects of movement. In our study, it seems that the spatial precision of the shift movement was given precedence over temporal control of movement during the high notes, due to a narrower pitch window on these notes (Chen et al., 2013). In other words, the participants may have lowered the speed of shift movements for the higher notes as an adaptive strategy to maintain the pitch accuracy of the shift movement. In contrast, temporal deviation between arrival at high notes and their respective metronome beats was reduced in the presence of the panel. With regard to the postural changes of the performers, it has been proposed that appraisals of danger leads to behavioral avoidance, characterized by retraction of the limbs toward own body (De Silva and Bianchi-Berthouze, 2004) and “freezing” them (Azevedo et al., 2005). From a behavioral perspective, the postural change observed in the current study may indicate a hint of upper limb withdrawal in a stressful environment. Furthermore, the resulting joint stiffness and reduced degrees of freedom reflect inefficient motor control and are typically associated with amateur performance (Verrel et al., 2013a). Postural changes observed in those who reported high in distress scores may underlie the regression of motor skills by the experienced cellists.

Although arousal lacks a universally accepted definition (Zaichkowsky and Baltzell, 2001), it is commonly seen as a state of heightened physiological activity, and the physiological responses are not largely different between high trait socially anxious and low trait socially anxious individuals (Mauss et al., 2004). Thus, increased physiological activities may reflect the state of arousal rather than anxiety, and previous research suggested that a stressful environment may improve performance by increasing motivation and effort of the actor (Hardy and Hutchinson, 2007). In support of this, in the present study, a detailed analysis on the elbow angle on completion of each shift movement revealed individual differences in how the physiological and behavioral measures are correlated; those participants with a high distress score showed a tendency to flex their left arm more when the heart rate was high but the reverse was true for those with a low distress score. Thus, heightened physiological activities could relate to dichotomous behavioral responses and perhaps, at least at a moderate level, stress can be beneficial for performance assuming that a more open left elbow angle helps to facilitate the shift movements. This observation is partly in line with predictions of multi-dimensional models such as the Yerkes-Dodson law (Yerkes and Dodson, 1908) and cusp catastrophe model (Hardy and Fazey, 1987), which postulate that the physiological arousal leads to small continuous changes in performance efficacy in the shape of an inverted *U*, and that a moderate level of arousal underlies optimal performance. Nevertheless, our study yields a more complex interaction between the physiological arousal and behavior which relationship is dynamically moderated with other factors as highlighted by Kenny (2011, pp. 141–143).

The design of the present study was shown to be valid with the participants reporting an increased level of distress whilst performing in front of the panel as measured by the subjective rating. The lack of difference in the subjective rating before and after a trial suggested that it is unlikely the participants reported their distress level based on the quality of the performance as a *post-hoc* deduction. This observation was further substantiated by the increased heart rate when the panel was present. In addition, the within-trial variation in heart rate reflected the difficulty of the performance, peaking at the time when they produced the most challenging high note (i.e., HN3). A similar within-trial fluctuation was observed in the EDA measure after accounting for its retarded response latency. The validity of our subjective scale for measuring the degree of anxiety is open to question, however. In our subjective measure, the Likert scale consisted of two-dimensional poles (unbearably anxious—completely relaxed), and the participants may have found it challenging to distinguish between anxiety and arousal. We attempted to strengthen the plausibility of our subjective measure by cross-validating it with standardized questionnaires. To this end, participants were asked to fill out two other questionnaires. We found that our subjective ratings were positively correlated with the *worry* and *tension* factors of the adapted Reactions to Tests questionnaire (Sarason, 1984), and the *Catastrophizing* factor in the Steptoe questionnaire (Steptoe and Fidler, 1987), suggesting at least some degree of agreement with other scales of subjective anxiety. Furthermore, our study could have been strengthened by seeking to investigate the relationship between the participants' response systems (subjective, behavioral and physiological) and their appraisal of the performance environment. A better understanding of how each participant evaluated the performance event in terms of goal relevance, goal congruence and ego-involvement (see Lazarus, 1991) might have thrown interesting light on what differentiated the group with positive outlook from that with a more negative outlook in terms of appraisal. We would recommend that future studies adopt this approach in order to discern a core relational theme for MPA.

In conclusion, we presented the kinematic changes exhibited by cellists when performing under pressure and their relationships with subjective and physiological responses. Our results highlight the interaction of the three components and the importance of understanding both subjective and physiological changes in musicians before attempting to predict the behavioral manifestations of performing in a stressful environment. However, it remains unclear as to what extent our findings are generalizable to the behavior of those who are clinically diagnosed with PA or in a significantly more anxiety-provoking environment such as at a real concert. As we present findings similar to those of Craske and Craig (1984), namely that physiological arousal is not necessarily concordant with anxious behavior in musical performance, we would argue that there is a case for encouraging an understanding of the concept of *performance arousal* in its physiological sense, as distinct from PA, in research and music education circles. This clarity of terminology would aid teachers in their efforts to explain that physiological arousal experienced before and during a performance is not always associated with the deleterious effects of anxious behavior, and that in players with a positive outlook such arousal may indeed be beneficial. Further understanding of how the different elements of MPA interact to affect music-making is essential for progress in the development of techniques and therapeutic interventions designed to help those who suffer from it, and other forms of PA.

### Conflict of interest statement

The authors declare that the research was conducted in the absence of any commercial or financial relationships that could be construed as a potential conflict of interest.
